# Glycopolymer Code Based on Well-Defined Glycopolymers or Glyconanomaterials and Their Biomolecular Recognition

**DOI:** 10.3389/fbioe.2014.00039

**Published:** 2014-10-14

**Authors:** Gokhan Yilmaz, C. Remzi Becer

**Affiliations:** ^1^Department of Chemistry, University of Warwick, Coventry, UK; ^2^Department of Basic Sciences, Turkish Military Academy, Ankara, Turkey; ^3^School of Engineering and Materials Science, Queen Mary University of London, London, UK

**Keywords:** glycopolymers, glycoparticles, well-defined polymers, biological functionalities, recognition events and drug delivery system

## Abstract

Advances in the glycopolymer technology have allowed the preparation of more complex and well-defined glycopolymers/particles with several architectures from linear to globular structures (such as micelles, dendrimers, and nanogels). In the last decade, functionalized self-assembled/decided nano-objects and scaffolds containing glycopolymers were designed to develop many biological and biomedical applications in diseases treatments such as pathogen detection, inhibitors of toxins, and lectin-based biosensors. These studies will facilitate the understanding and investigation of the sugar code on the carbohydrate–lectin interactions, which are significantly influenced by the glycopolymer architecture, valency, size, and density of binding elements. In this context, these advanced and selected glycopolymers/particles showing specific interactions with various lectins are highlighted.

## Introduction

Carbohydrates differ from other biological macromolecules in terms of their monomeric units and their ability to be highly branched molecules (Gabius et al., 2004; Ambrosi et al., 2005a; Rudiger et al., 2010). Polymer chemists and biologists are still studying to answer two critical questions, which are the foundations of sugar coding (i) How do carbohydrates influence the properties of the lectins to which they are attached? (ii) How do carbohydrates get involved in recognition events? Oligosaccharides have a high-density coding capacity due to the variations in anomeric status, linkage positions, ring size, branching, and introduction of site specific substitutions (Gabius et al., 2002). Their major function is to serve as recognition markers (Gamblin et al., 2009). Large range of oligosaccharides may allow them to cover functionally important areas of lectins, to modulate the interactions of glycoconjugates with other molecules, and to affect the rate of processes, which involve conformational changes due to the very sensitive sugar coding. This special sugar coding system let them to have crucial biological roles with unusual oligosaccharide sequences, unusual presentations of common terminal sequences, and further modifications of the sugars themselves. However, the investigation on their functionalities is rather limited due to the difficulties in synthesizing such structures. Although there have been major developments to chemically synthesize oligosaccharides, such as introduction of good anomeric leaving groups and advances in the solid-phase synthesis techniques, it is still demanding to isolate, purify, and analyze the complete structure of oligosaccharides (Bertozzi and Kiessling, 2001).

Glycopolymers, which are synthetic macromolecules with sugar moieties, have been considered as alternative structures to oligosaccharides (Kiessling et al., 2006). They generally exhibit a crucial role for many biological processes such as signal transmission, intercellular recognition, and fertilization (Ladmiral et al., 2004; Granville et al., 2007; Spain et al., 2007; Deng et al., 2009; Hetzer et al., 2010; Schubert et al., 2011; Slavin et al., 2011; Spain and Cameron, 2011; Gou et al., 2012a; Shi et al., 2012). Although the interaction between carbohydrates and lectins is weak, it could be greatly enhanced by the multivalent effect of densely packed carbohydrate molecules with unique functionalities, which is known as the “glycocluster effect” (Mateescu et al., 2009; Ting et al., 2010). Hence, understanding and investigation of this specific interaction between glycopolymer and protein with high selectivity and strength are becoming more attractive for polymer chemists due to their potential ability for useful in biomimetic applications and human therapeutics (Geng et al., 2007; Ruiz et al., 2008; Mateescu et al., 2009). Despite of the fact that many different types of glycopolymers including linear and spherical glycopolymers, glycodendrimers in the form of micelles, vesicles, and micro/nanoparticles have been prepared by now, it is still challenge to synthesize precision glycopolymers with different chain lengths, compositions, and architectures (Ladmiral et al., 2006; Narain, 2006; Geng et al., 2007; Chen et al., 2010; Min et al., 2010; Becer, 2012). However, some recent studies have shown a sufficient control in chain length, architecture, monomer sequence, chain folding, and tertiary structures to achieve the synthesis of precision glycopolymers (Hartmann et al., 2006; Semsarilar et al., 2010; Voit and Appelhans, 2010; Börner, 2011; Geng et al., 2011; Schmidt et al., 2011; McHale et al., 2012; Zamfir and Lutz, 2012). Generally, there are two advanced synthesis methods to prepare well-defined glycopolymers, which are polymerization of carbohydrate-bearing monomers without the deprotection steps and chemical modifications of functional polymers with carbohydrates via controlled/living polymerization techniques in combination with click reactions. According to these promising recent approaches on the preparation of sequenced glycopolymers as well as understanding their interactions with lectins, we believe chemists will be able to contribute to the design of the new generation sequence controlled glycopolymers with multiple functionalities incorporated with a nanometer precision.

In this review article, we initially focused on these precision glycopolymers having relatively good control over the sequence and their binding affinity with different lectins (Dong et al., 2003, 2004; Geng et al., 2008; Miura et al., 2008; Xiao et al., 2008; Becer et al., 2009; Chen et al., 2009; Nurmi et al., 2009; Thomas and Lutz, 2011). Secondly, the most commonly used analytical techniques for the measurement of the carbohydrate–lectin binding were discussed. And then, general recent developments on the synthesis of glycopolymers and glyconanoparticles, and their recognitions with lectins were highlighted.

## Precision Synthesis of Glycopolymers

Controlled and living polymerization techniques in combination with using different routes such as click reactions and grafting to surface allowed to prepare precision glycopolymers of predetermined weight and narrow molecular weight distribution, as well as various block copolymers and a wide range of complex macromolecular architectures. The main most used controlled/living radical polymerization techniques for well-defined glycopolymer, nitroxide-mediated polymerization (NMP), atom transfer radical polymerization (ATRP), reversible addition-fragmentation chain transfer (RAFT), and single electron transfer-living radical polymerization (SET-LRP) and ring-opening polymerization (ROP) are relatively more tolerant to prepare well-defined glycopolymers with different functionalities (Lele et al., 2005; Chen et al., 2007; Narain et al., 2007; Boyer and Davis, 2009; Toyoshima and Miura, 2009; Becer et al., 2010; Godula and Bertozzi, 2010; Yu and Kizhakkedathu, 2010; Belardi et al., 2012; Wang et al., 2012; Ahmed and Narain, 2013; Ahmed et al., 2013). Moreover, the most important advantage of these techniques is their applicability to use a very wide range of monomers under a large number of experimental conditions. During the last couple of years, the explosive number of publications devoted to prepare glycopolymers via these polymerization techniques that have a binding ability with different lectins prompted us to contribute this updated review.

### Sequence control based on solid-phase synthesis approach

**Figure 1:**
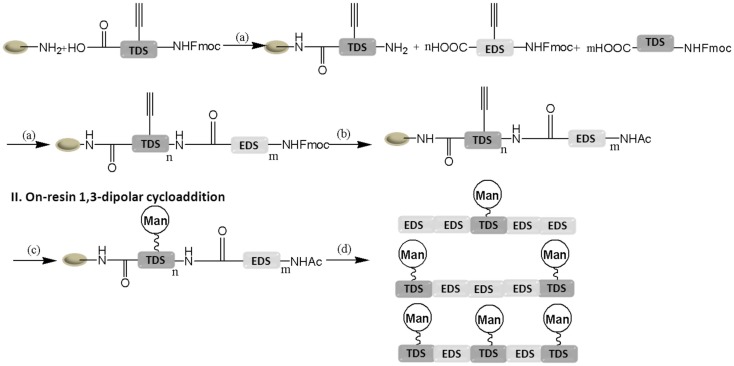
**Solid-phase synthesis of glycopolymer segments (Ponader et al., 2012)**.

Ponader et al. (2012) have firstly reported the synthesis of sequence-defined monodisperse glycopolymer segments by a combination of solid-phase synthesis and click chemistry. The triple bond functionalized building block 1-(Fluorenyl)-3,11-dioxo-7-(pent-4-ynoyl)-2-oxa-4,7,10-triazatetradecan-14-oic acid (TDS) and ethylenedioxy (EDS) building block from a commercially available diethylenetriamine and 2,2′ (Ethylenedioxy)bis (ethylamine) were prepared, respectively, in several steps. These building blocks having an fluorenylmethyloxycarbonyl (Fmoc)-protected amine to build block coupling on solid-phase according to standard Fmoc SPPS protocols allow for the sequence-defined positioning of alkyne moieties within monodisperse poly(amidoamine) PAA segments. Trityl-tentagel-OH resin modified with ethylenediamine linker used as a solid support for the coupling reaction of building blocks TDS and EDS on the resin (Figure 1).

In order to perform the click reaction, the alkyne group of the PAA backbone was deprotected by removal of the Fmoc protecting group with a 25% solution of piperidine in DMF. The click reaction between these alkyne groups and azidoethyl mannosides was performed using sodium ascorbate and CuSO_4_ in the solvent mixture of DMF and water. After cleaving the short chain from the support resin, three different monodisperse glycopolymer segments were obtained with the same contour length but differ in their number and spacing of presented mannose ligands. Three different precision short glycopolymer chains having the same contour length but differ in their number and spacing of presented mannose ligands were obtained after cleaving from the support resin. As expected, trivalent glycopolymer segment showed higher binding affinity than others due to the number and position of mannose. Although this strategy provides to synthesis precision glycopolymers with the desired chain length, structure, and composition, it is still challenge to prepare long-chain glycopolymers with a good sequence control via this method.

### Sequence control based on the reactivity ratios of comonomers

**Figure 2:**
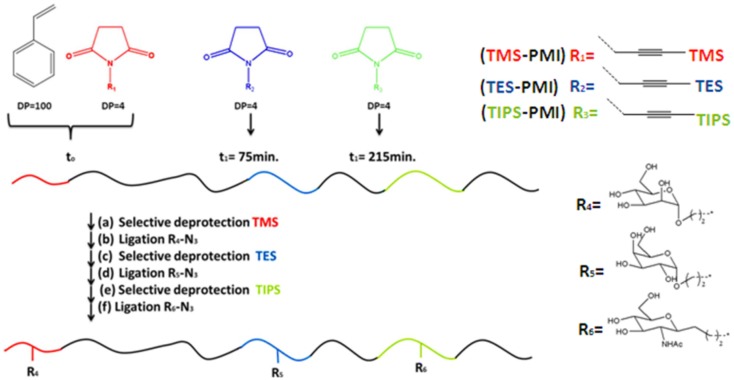
**General strategy for the synthesis of single-chain sugar arrays**. Experimental conditions: (a) K_2_CO_3_, MeOH/H_2_O/THF, 40°C, 7 h; (b) CuBr, 4,4′-di-*n*-nonyl-2,2′-bipyridine, DMF, room temperature; (c) K_2_CO_3_, MeOH/H_2_O/THF, 60°C, 89 h; (d) TBAF, THF, room temperature, overnight. DMF, *N,N*-dimethylformamide, NMP, nitroxide-mediated polymerization; TBAF, tetrabutylammonium fluoride (Baradel et al., 2013).

An elegant approach to prepare precision glycopolymers in a convenient manner was reported by Baradel et al. (2013). They have utilized a combination of “living” radical polymerization technique and copper-catalyzed azide–alkyne cycloaddition (CuAAC). The NMP was performed to yield copolymers of styrene and maleimides (MIs) derivatives. Polymerization kinetics of these monomers showed a marked influence on macromolecular sequences and lead to efficient sequence controlled. The polymerization reaction was carried out in anisole at 120°C using alkoxyamine BlocBuilder as an initiator. The time-controlled addition of the protected MIs (three equivalents) during the polymerization of styrene, provided linear polystyrene chains presenting short but localized glycol functional regions (PDI = 1.23–1.24). Initially, triisopropylsilyl protected *N*-propargylmaleimide (TIPS-PMI) monomer was polymerized at the beginning of the reaction and then triethylsilyl protected *N*-propargylmaleimide (TES-PMI) was introduced after reaching full conversion of TIPS-PMI and further chain growth with styrene. Finally, trimethylsilyl protected *N*-propargylmaleimide (TMS-PMI) was added close to the end of the polymer chain due to their potential sensitivity to the reaction conditions (Figure 2). The precise chain positioning of all three reactive MIs was confirmed by the study of the kinetics of copolymerization.

Following the synthesis of precision polymers with controlled molecular weights and molecular weight distributions, the stepwise orthogonal deprotection and functionalization of the three distinct alkyne sites with different azide-functionalized sugars were studied. Firstly, the deprotection reaction for the removal of TMS group was carried out in a methanol/water/THF mixture with K_2_CO_3_ at 40°C for 7 h, and then the azidomannose was attached to the polymer via CuAAC click reaction. The TES group was then removed by treatment with K_2_CO_3_ in a methanol/water mixture at 60°C for 89 h, and then the alkyne was reacted with the azido-galactose derivative. Lastly, the last protecting TIPS group was removed in THF with tetrabutylammonium fluoride at room temperature for overnight and the clickable alkyne site reacted with the azido-*N*-acetylglucosamine derivative. The click reactions can be followed by the appearance of broad signals in the region δ = 3.1–5.0 ppm in ^1^H NMR corresponding to the attachment of sugar groups. All click reactions were carried out in DMF at room temperature using of Cu(I)Br and 4,4′-di-*n*-nonyl-2,2′-bipyridine as the catalyst system.

The analyze of the binding ability of the modified glycopolymers to hexose-specific lectins, namely, concanavalin A (ConA), peanut agglutinin (PNA), and wheat-germ agglutinin (WGA), was done by using the quartz crystal microbalance with dissipation monitoring (QCM-D) technique. Interestingly, these lectins showed high-binding affinity with mannose, galactose, and *N*-acetylglucosamine groups, respectively. This work is critically important to understand the complexity of GlycoCode (Becer, 2012) and lead other research groups to the synthesis of complex precision glycopolymers as a glycan mimic.

### Sequence control based on sequential addition of monomers

**Figure 3:**
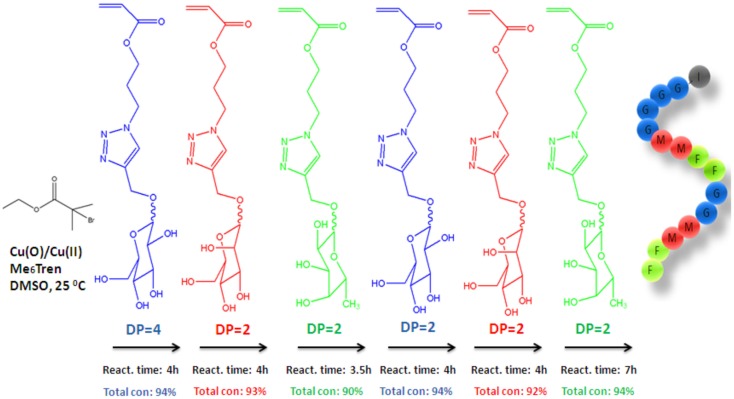
**Schematic representation of the synthesis of multiblock glycopolymers by iterative addition of glycomonomers at defined time period (Zhang et al., 2013)**.

A versatile method to prepare different types of precision multi-block glycopolymers was performed by Zhang et al. (2013) via controlled polymerization technique, namely, SET-LRP. Firstly, several types of glycomonomers were prepared by clicking of 3-azidopropylacrylate (APA) to alkylated mannose, glucose, and fucose, respectively, via the CuAAC click reaction with CuSO_4_ and sodium ascorbate in methanol/water mixture. Initially, the polymerization of glucose monomer (GluA) was undertaken using Cu(0)/Cu(II)/*tris*[2(dimethylamino)ethyl]amine (Me_6_TREN) as the catalyst system in DMF at 20°C and yielded high-chain end fidelity glycopolymer allowed for polymerization of more of the same or different glycomonomers in one pot. As depicted in Figure 3, chain extension was carried out by addition of mannose monomer (ManA) in 1 ml DMSO into the polymerization reaction mixture via cannula under nitrogen. After reaching full monomer conversion, fucose monomer (FucA) was added with a same way. This sequential addition of the subsequent monomers was repeated one more time and continued until the glycopolymer reached to six short blocks (DP = 2 for each block; Mn = 7.6 kDa; Mw/Mn = 1.13). Moreover, the polymerization of di(ethylene glycol) ethyl ether acrylate (DEGEEA) and ManA was developed for a model thermoresponsive sequence controlled multi-block glycopolymers in a relatively large scale until reaching six blocks (Mn = 69.6 kDa; Mw/Mn = 1.23).

This work was the first to report the investigation of the binding affinity of the highly sequence controlled glycopolymers with the human lectin dendritic cell-specific intercellular adhesion molecule-3-grabbing non-integrin (DC-SIGN). Their lectin binding properties was measured via SPR. Despite of the fact that the binding ability of the resulting precision glycopolymers exhibited a clear dependence with the number-average degree of polymerization of each glycomonomer, the glycopolymers with different sugar units did not present enough binding affinity, as well.

As opposed to the direct polymerization of glycomonomers, an alternative route was employed to prepare for precision multi-block glycopolymers by using the combination of copper(0) mediated living radical polymerization [Cu(0)-LRP] with thiol-halogen, thiol-epoxy and CuAAC click chemistry (Zhang et al., 2014). Well-defined alkyne and epoxide side-chain polymeric scaffold were obtained by polymerization of glycidyl acrylate (GA) and acrylic acid 3-trimethylsilanyl-prop-2-ynyl ester (TMSPA) from Cu(0)-LRP using Cu(0) wire as the activator and additional CuBr_2_ as the deactivator and Me_6_TREN as the ligand in DMSO at ambient temperature, allowed for further sequential thiol-halogen, thiol-epoxy, and CuAAC reactions to build functional glycopolymers with a defined sequence and spatial orientation. The reactivity of these glycopolymers with appropriate lectins has not been demonstrated yet.

All these reports on the synthesis of precision glycopolymers prepared by different techniques are quite promising in mimicking structural and functional responsibilities of glycocalyx (Yilmaz and Becer, 2013). They are versatile examples to take this challenge in the polymer science one more step forward and lead other research groups to synthesis controlled folding polymers having a great potential to be utilized in biological applications. Although the polymerization of monomers bearing carbohydrate can be carried out by a range of polymerization techniques, it is not suitable for introducing some different functional groups along polymer backbones. It should be mentioned that these reports opened new avenues for the synthesis of complex precision glycopolymers. SET-LRP technique was used to build multi-block glycopolymers with short blocks of sugar monomers in one pot by sequential addition of the subsequent monomers in a relatively large scale (Zhang et al., 2013, 2014). Unfortunately, it was not possible to determine any effect of sequence on binding in the system due to low degree of polymerization of each glycomonomer (DP = 2).

A metal-free living radical polymerization technique, NMP, was used for incorporation of the desired sugar units at the designed location of the chain. Although NMP represents an excellent avenue for the polymerization of styrene and its derivatives having bioinert and biocompatible properties, it does not show the tolerance to the flexible range of reaction conditions in terms of the solvents, monomers, and temperatures.

Last but not least, solid-phase synthesis allowed achieving an excellent sequence control with the low quantity of the isolated material after several steps. Despite these recent developments, it is still essential to advance precision sequence controlled synthesis techniques to encode glycopolymer–lectin binding code and to prepare more complex precision glycopolymers as a glycan mimic. These studies provided not only new routes to prepare precision glycopolymers that only differ from each other for the length and the nature linker connecting the sugar units to the macromolecular backbone but also to compare structurally similar ligands that only differ for the nature of the carbohydrate moieties.

## Analytical Techniques to Determine Glycopolymer–Lectin Interactions

Various analytical methods have been developed to study multivalent carbohydrate–lectin interactions (Smith et al., 1968; Matrosovich et al., 1990; Islam Khan et al., 1991; Roy et al., 1998; Burke et al., 2000). The most widely applied and oldest technique is turbidimetry and based on the determination of the turbidity of the solution up on aggregation of lectin and polymer chains. UV–Vis spectrometer can be utilized to determine the turbidity of the solution at varying ratio of lectin to glycopolymers (Wagner et al., 2002). Alternatively, quantitative precipitation and quenching of fluorescence emission techniques have been employed to distinguish effect arising from receptor clustering and receptor proximity, respectively (De Clercq, 2006; McReynolds and Gervay-Hague, 2007). More advanced techniques, such as isothermal titration calorimetry (ITC), atomic force microscopy (AFM), quartz crystal microbalance (QCM), and surface plasmon resonance (SPR) spectroscopy have also been widely used to investigate carbohydrate–lectin interactions (Sauerbrey, 1959; Homola et al., 1999; Zlatanova et al., 2000; Marx, 2003; Ambrosi et al., 2005b; Chiad et al., 2009). This section will briefly describe these techniques with the selected examples and their benefits and disadvantages.

**Figure 4:**
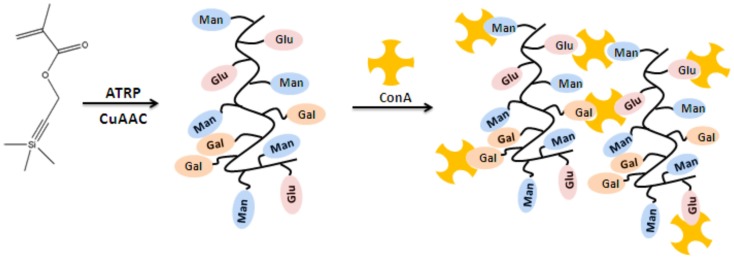
**Schematic representation of the glycopolymer lectin binding (Gou et al., 2013)**.

A library of well-defined glycopolymers with different sugar azides (mannose, galactose, and glucose) and their ability to form cluster with ConA via five different assays analysis (quantitative precipitation, turbidimetry, fluorescence quenching assays, inhibitory potency assays, reversal aggregation assays) have been reported very recently (Gou et al., 2013). ConA was chosen as a model lectin due to the interactions with a wide range of saccharides, especially mannose and glucose. The influence of the nature and densities of different sugars residues on the stoichiometry of the cluster, the rate of the cluster formation, the inhibitory potency of the glycopolymers, and the stability of the turbidity were investigated via using these different assays. The glycopolymer that was substituted with one mannose residue per repeat unit showed the highest and strongest binding ability as multivalent ligand for clustering ConA (Figure 4). Therefore, mannose residues seem to be the main factor to enhance multiple interactions for the binding stoichiometry, the rate of binding, the potency, and the stability of ConA clustering. The other factor is that an increasing the chain length provided enhanced binding, but it was less benefit after a certain length. Due to the results, this work is a crucial example to demonstrate that how the well-defined glycopolymers with various pendant epitopes have a big influence on the lectin-multivalent interaction and the stability of glycopolymer-ConA cluster regarding the changing of cluster formation parameters.

It was possible to measure the sedimentation velocity related to the binding rate of lectins and carbohydrates by using more advanced technique such as analytical ultracentrifugation. Moreover, the thermodynamics of binding between lectins and multivalent ligands could be determined from ITC data by using and appropriate binding model. Single molecule force spectroscopy has recently been developed into a highly sensitive tool for the investigation of single biomolecule interactions (Chiad et al., 2009). Alternatively, atomic force microscopy (AFM) or optical tweezers has been used to measure dissociation forces of single ligand–receptor complexes in the piconewton range (Zlatanova et al., 2000).

Quartz crystal microbalance is a simple, cost effective, high-resolution mass sensing technique, which has been employed to study a wide range of molecular systems at the solution–surface interface (Marx, 2003). QCM with dissipation monitoring (QCM-D) technique is a special type of QCM based on the ring-down technique relevant to the piezoelectric effect in quartz crystals, whose frequency of oscillation changes in proportion to the amount of mass adsorbed onto their surface. Up to four parallel measurements can be performed in selected QCM-D instruments. However, the main disadvantages of this technique is that it requires 10–100 mg of sample and relatively long time, typically a few hours, to reach the equilibrium in binding.

**Figure 5:**
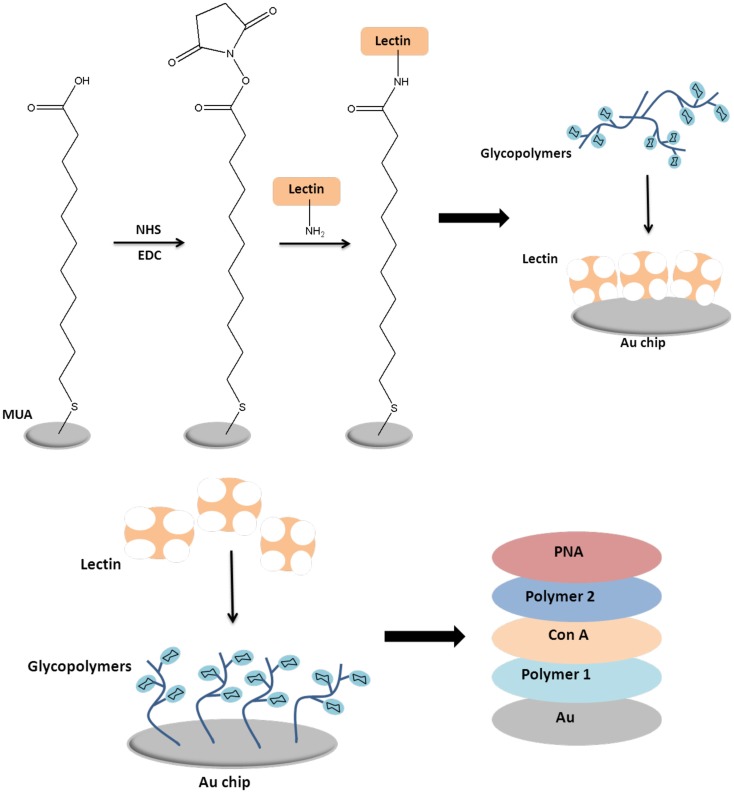
**Formation of layer-by-layer assembly using quartz crystal microbalance technique (Gou et al., 2012b)**.

As illustrated in Figure 5, layer-by-layer (LBL) formation of glycopolymer and lectin was performed using QCM-D (Gou et al., 2012b). As the first step, gold chip was chemically modified with 11-mercaptoundecanoic acid (MUA), followed by 1-[3-(dimethylamino)-propyl]-3-ethyl carbodiimide (EDC) hydrochloride and *N*-hydroxysuccinimide (NHS). Subsequently, ConA was bound to the surface via nucleophilic substitution of lysine and ethanolamine hydrochloride was used to block unreacted NHS groups to prevent their interaction with glycopolymers. Mannose glycopolymer was flown over the surface to determine the rate of binding. The sufficient binding was achieved because of the existing galactose units in the glycopolymer. In the second part of the study, Becer et al. reported the LBL formation of glycopolymers and different lectins for the first time. A mannose-containing glycopolymers with a disulfide linkage positioned in the middle of the chain was reacted with the bare gold surface. Following to this, ConA was flown over the surface to react with the first layer of mannose glycopolymer. Then, a glycopolymer that is composed of both mannose and galactose units were utilized. The mannose units were bound to free binding sites of ConA and free galactose units were used to bind to PNA that was flown subsequently. Therefore, a sandwich of mannose polymer-ConA-mannose/galactose copolymer-PNA has been formed on gold surface. This approach has a potential to be used in glycoparticles that could be utilized in targeted drug delivery applications.

Surface plasmon resonance spectrometer monitors the interaction of two or more molecules or molecular assemblies in real time. Basically, SPR is based on the measurement of adsorption of material onto planar metal surfaces (Homola et al., 1999). SPR utilizes the flow of analyte solution over a functionalized gold surface, resulting in a change in the refractive index. This technique is highly sensitive and can be used to detect association of glycoproteins or glycopolymers in pico-molar concentrations. In a recent report, SPR has been utilized to investigate the interaction of mannose-containing glycopolymers with a dendritic cell lectin, DC-SIGN, and the ability of these glycopolymers to inhibit the interactions between DC-SIGN and HIV envelope glycoprotein, gp120 (Becer et al., 2010). Multi-channel surface plasmon resonance (MC-SPR) was used to investigate the binding affinity of a library of glycopolymers with bacterially expressed soluble recombinant human DC-SIGN tetramers (Becer et al., 2010). DC-SIGN was immobilized onto an SPR sensor chip and the interactions between DC-SIGN and the glycopolymers were probed as a function of glycopolymer concentration. As expected, homopolymer of mannose exhibited the highest binding affinity due to highest concentration of mannose groups per polymer chain. The main advantages of SPR are that it requires extremely low amount of sample and the multi-channel system can detect 36 interactions in parallel in <30 min of run time.

In summary, several successful analytical techniques have been developed to study multivalent carbohydrate–lectin interactions. In particular, QCM and SPR techniques are highly sensitive in detection and provide more reliable data because the ligands or analytes can be immobilized on a surface. Indeed, there is a need for combination of different techniques and development of precisely sequence controlled synthesis techniques to have a better understanding in glycopolymer–lectin binding activities and to establish the synthetic glycopolymer code.

## Functional Glycomaterials and Their Applications

### Glycopolymers with different carbohydrate linkers

**Figure 6:**
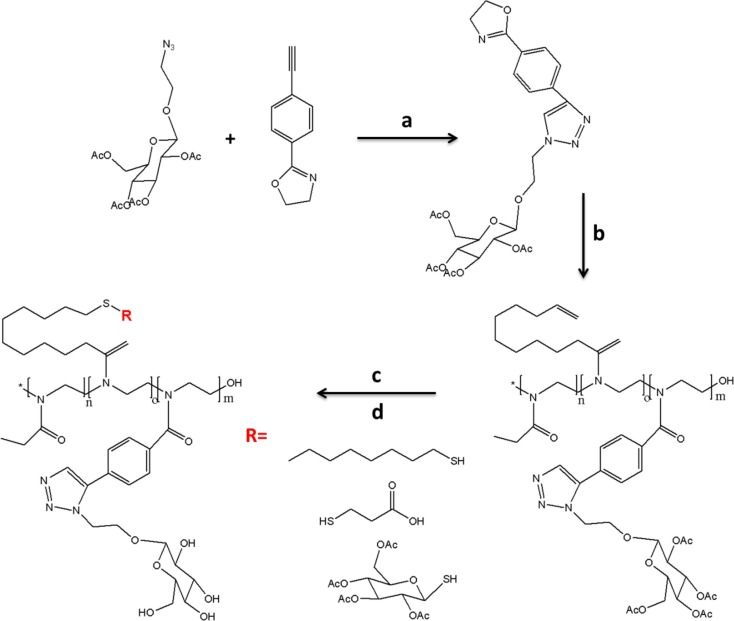
**Schematic presentation of the synthesis glycopolymer: (a) CuAAC click reaction was carried out in THF/H_2_O (5:1) with sodium ascorbate and copper(II) acetate; (b) copolymerization was undertaken in the microwave at 120°C for 12 h to reach full conversion of all three monomers; (c) thiol-ene photoaddition reaction by using different thiols; (d) deprotection was done in the presence of sodium methanolate in dry dichloromethane (Kempe et al., 2011)**.

Kempe et al. (2011) have employed the synthesis of a new sugar functionalized 2-oxazoline monomer (Figure 6). In order to prepare this glycomonomer, 4-bromobenzonitrile was reacted with 2-aminoethanol in the presence of a Lewis-acid catalyst. The alkyne groups were modified with a trimethylsilyl (TMS) protection group. The glycomonomer, 2-(azidoethyl)-1-β-d-glucopyranosetetraacetate (Ac_4_Glc-YnOx), was obtained with high yield via CuAAC reaction using Cu(II) acetate and sodium ascorbate after the removing TMS groups.

The copolymerization of Ac_4_Glc-YnOx with 2-ethyl-2-oxazoline and 2-(dec-9-enyl)-2-oxazoline was carried out via the microwave synthesizer using methyl tosylate as an initiator in acetonitrile at 120°C for 12 h. After that, the pendant alkene group in the side-chain of these well-defined glycopolymers was modified with different thiols, namely, 2,3,4,6-tetra-*O*-acetyl-1-thio-β-d-glucopyranose, dodecanethiol, and 3-mercaptopropionic acid via thiol-ene photoaddition reaction to analyze thermosensitivity and pH sensitivity of these glycopolymers in water and PBS. Finally, these glycopolymers showed strong binding ability with ConA.

A synthetic methodology based on three tandem post-polymerization and modifications to synthesize glycopolymers with high specificity and selectivity as mimicking glycan architecture due to secondary binding motif over the polymer side-chain has been reported (Jones et al., 2014). Initially, glycidyl methacrylate (GMA) was polymerized via Cu(I)-mediated polymerization and introduced with sodium azide in DMF at 50°C to yield the azide functional groups. The reaction produced a secondary alcohol that was followed by infrared spectroscopy (IR). Subsequently, acyl chlorides were reacted with alcohol to install secondary motifs due to the fact that aromatic groups can bind to the sialic acid site. Following to this modification, the azide groups were reacted with β-d-propargyl galactose by Cu(I)-catalyzed cycloaddition. By this route, a small library of glycopolymers was prepared and used to study their binding affinity to their corresponding lectins. The bacterial toxin (CTx), the causative agent of cholera infection, and PNA, a model galactose-binding non-pathogenic lectin, were used for the investigation of the binding affinity of these glycopolymers. According to the assay studies, the specificity and selectivity of the glycopolymers to CTx increased with a secondary binding pocket within CTx due to allosteric interactions of the neuraminic acid in the CTx. Generally, PNA showed higher binding ability than CTx. This work achieved to use glycan-mimetic branching to introduce specificity and selectivity as well as affinity into synthetic glycopolymers.

### Fluorescently labeled glycopolymers

**Figure 7:**
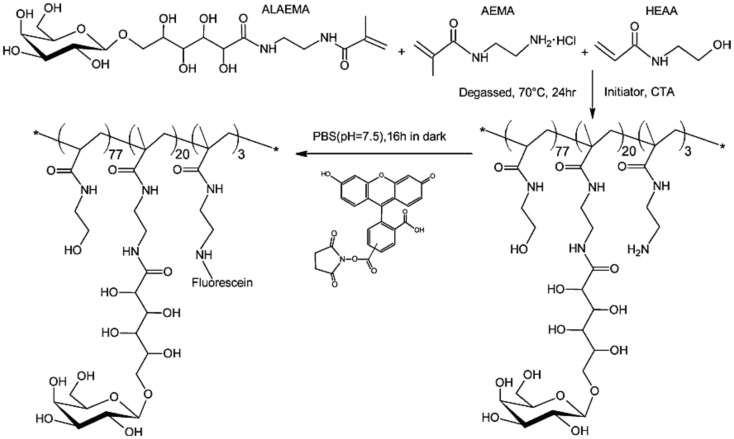
**Illustration of the synthesis of fluorescent glycopolymers PMA-ALAEMA-Fluorescein containing β-galactoside as the pendant sugar**. Reproduced with permission from Springer, Copyright 2013 (Wang et al., 2014a).

Wang et al. (2014a) developed RAFT-based one-step polymerizations to prepare tri-component statistical fluorescent glycopolymers as illustrated in Figure 7. Three different monomers including *N*-(2-hydroxyethyl) acrylamide (HEAA), *N*-(2-aminoethyl)methacrylamide (AEMA), and *N*-(2-glyconamidoethyl)-methacrylamides possessing varied pendant carbohydrates were used for the one-step tri-component RAFT-based copolymerization. Totally, five different glycomonomers were synthesized by using the same procedure. RAFT copolymerization was carried out in DMF using (4-cyanopentanoic acid)-4-dithiobenzoate as a RAFT agent and 4,4′-azobis-(4-cyanovaleric acid) as an initiator at 70°C for 24 h. Their subsequent modification with varied fluorescent labels due to functional primary amine groups on AEMA afforded a well-defined statistical tri-component glycopolymers with varied fluorescent labels demonstrating versatile reporters of specific lectins.

*Pseudomonas aeruginosa* lectin (PA-IL) bacteria and *Galanthus nivalis* plant lectin (GNL) were used to analyze the binding ability of these tri-component glycopolymers. While the α-mannose-containing polymer showed very strong binding with GNL, β-d-galactose-containing polymer showed enhanced binding ability with PA-IL. This report presented a new way to prepare a wide range of tri-component glycopolymers via RAFT-based one-pot polymerization.

### Glycopolymer bioconjugates

**Figure 8:**
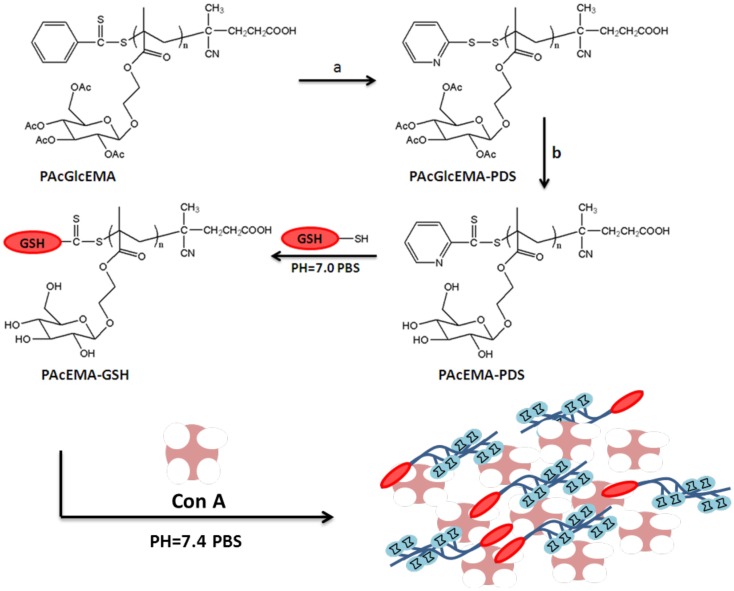
**Schematic presentation of the synthesis of glycopolymer–peptide bioconjugate PGlcEMA-GSH via RAFT polymerization and thiol–disulfide exchange**. Reagents and conditions: (a) 2,2′-dithiodipyridine, ethanolamine, acetonitrile, room temperature, 24 h; (b) sodium methoxide, CH_3_Cl/MeOH (1:1), room temperature, 1 h (Shi et al., 2012).

Shi et al. (2012) reported the synthesis pyridyldisulfide (PDS) functional well-defined glycopolymer by RAFT polymerization of 2-(2,3,4,6-tetra-*O*-acetyl-β-d-glucosyl (oxy)ethyl methacrylate) (AcGlcEMA) glycomonomer in the presence of 4-cyanopentanoic acid dithiobenzoate (CPADB) and 4,4′-Azobis(4-cyanopentanoic acid) (ACPA) in 1,4-dioxane at 70°C for 24 h. The molar ratio of chain transfer agent to initiator was chosen as 10:1 and the polymerization was stopped at low monomer conversion (50–60%) in order to obtain efficient di-thioseter end functionalities for the subsequent modification with PDS. End-group modification was performed by aminolysis in the presence of ethanolamine and subsequently reaction with excess 2,2′-dithiodipyridine (DTP). After the removal of acetyl protecting groups, GSH was conjugated to PGlcEMA-PDS through the thiol–disulfide exchange reaction in 5 ml of phosphate buffer at pH 7.0 for 24 h under N_2_ at room temperature as shown in Figure 8. The reaction was followed via a UV–Vis spectrophotometer at 343 nm and the pure glycopolymer–peptide bioconjugate was confirmed by the disappearance of the significant signals of pyridyl ring protons at δ 7.4–8.5 ppm. The binding affinity of the peptide-glycopolymer bioconjugate with ConA was been studied via UV–Vis spectroscopy, fluorescence quenching titration, and transmission electron microscopy (TEM) indicating good level of sugar–lectin binding as expected. Furthermore, its antioxidant activity was investigated *in vitro* using 1,1-diphenyl-2-picrylhydrazyl (DPPH) assay. The obtained results showed that PGlcEM-GSH bioconjugates are promising for the development of antioxidant delivery system, biomimetics, and biodetection.

An elegant strategy, based on the work of Godula and Bertozzi (2012) regarding preparing a series of fluorescent mucin mimetics displaying a range of α-*N*-acetylgalactosamine (GalNAc) valencies, was developed for the construction of a mucin mimetic glycopolymer microarray. To synthesize a glycopolymer as mucin mimicking, methylvinyl ketone was polymerized via RAFT using biotin containing trithiocarbonate chain transfer agent and 4,4′-azobis(4-yanovaleric acid) (ACVA) as a radical initiator in 2-butonane at 65°C for 16.5 h. The obtained polymers had biotin terminal functional groups at their one end for further conjugation to streptavidin-coated microarray substrates. The opposite end of the polymer chains was functionalized with a maleimide-functionalized Cy3 dye because of their use as a fluorescent label. The last step was the synthesis of biotin-terminated glycopolymer with a Cy3 label and the reaction was carried out in the presence of α-aminooxy-GalNAc under acidic conditions at 50°C for 20 h. After the attachment of the biotinylated mucin mimetics to the array surface with ~40–60% efficiency, their binding ability with four lectins [soybean agglutinin (SBA), *Wisteria floribunda* lectin (WFL), *Vicia villosa*-B-4 agglutinin (VVA), and *Helix pomatia* agglutin (HPA)] was examined. Generally, while HPA showed stronger avidities than other lectins toward all the polymers irrespective of their GalNAc valency, SBA showed propensity to cross-link the high-valency mucin mimetics. Interestingly, increasing in surface density array did not show any significant enhancement for the binding affinity of all lectins.

### Amphiphilic block glycopolymers for self-assembled structures

**Figure 9:**
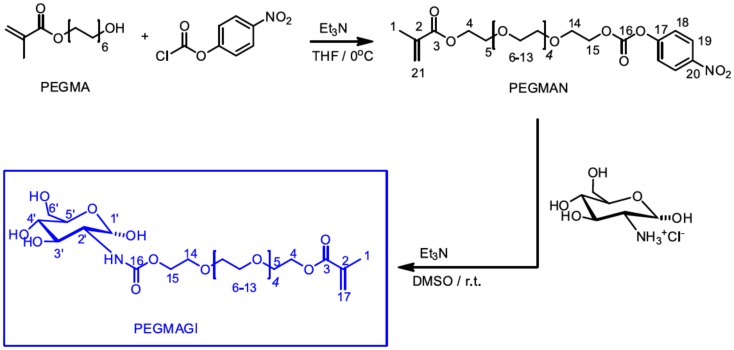
**Outline synthesis procedure of the glycomonomer, PEGMAGl**. Reproduced with permission from Elsevier, Copyright 2014 (Alvárez-Paino et al., 2014).

Alvárez-Paino et al. (2014) reported the synthesis of different amphiphilic glycopolymers as illustrated in Figure 9. Poly(ethylene glycol) methacrylate (PEGMA) was used to prepare a glycomonomer for further copolymerization methyl acrylate (MA) via free radical polymerization varying the initial feed composition. Firstly, PEGMA was activated with *p*-nitrophenylchloroformate in the presence of TEA in THF at 0°C for 24 h. After purification, the modified monomer with *p*-nitrophenyl carbonate groups was reacted with the glucosamine using TEA and hydroquinone in THF at room temperature to afford the glycomonomer. The copolymerization of MA and the obtained glycomonomer was carried out with azodiisobutyronitrile (AIBN) as an initiator in DMSO at 70°C. The different amphiphilic glycopolymers with the different ratio of the monomers were prepared by using this approach. The emulsion polymerization of MA was done with these glycopolymers as surfactants without the addition of any co-surfactant and the formation of colloidal stability, spherical, monodisperse, and small latex particles with low efficiency were achieved. The hydrophobic and insoluble part of these glycopolymers provided enhanced attachment of copolymer surfactant to the particles. Fluorescence microscopy was used to analyze the binding activity of these polymer-coated particles with ConA. The results showed the number of glycounits in the glycopolymer stabilizers affected the binding ability directly and significantly. Moreover, the films containing PEGMAGl-based copolymers showed higher binding affinity with ConA due to their flexibility.

**Figure 10:**
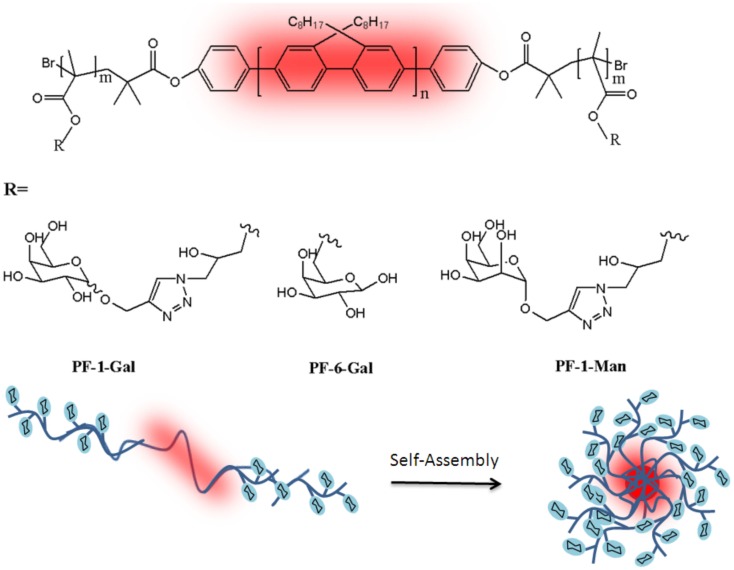
**Chemical structures of PF-1-Gal, PF-6-Gal, and PF-1-Man and schematic representation of the self-assembled nanoparticles (Sun et al., 2013)**.

In a similar approach, Sun et al. (2013) reported the effects of sugar regioisomerism (glycosidic linkage on different hydroxyl groups of the same sugar) on the binding ability of the self-assembled nanoparticles with lectins in a multivalent manner and the subsequent cell-uptake pathways as well. Basically, three self-assembled nanoparticles were prepared from triblock copolymers with the same polymeric backbone but different sugar regioisomers as pendant groups. Galactose was used as well-defined constitutional isomers due to its anomeric position (1-Gal) or 6-position (6-Gal) and a mannopyranoside (1-Man) as a control. Firstly, rod block of poly(9,9-dioctylfluorene) macroinitiator was conjugated with the bromine-functionalized polyfluorene (PF) initiator from the two ends of it for further occurring the middle block of the glycopolymers. Poly(glycidyl methacrylate) (PGMA) was synthesized via ATRP in the presence of the synthesized macroinitiator to yield triblock copolymer (PGMA-*b*-PF-*b*-PGMA). The pendent epoxide groups of the PGMA block were reacted with NaN_3_ for the sugars modified with alkynes click targeting. 1-(2′-Propargyl)-d-galactose (α:β = 10:3) and 1-(2′-propargyl)-d-mannose were prepared and used for the click chemistry that yielded PF-1-Gal, PF-6-Gal, and PF-1-Man (Figure 10).

These glycopolymers contain the same middle rod block and side coil blocks with similar DP were performed for their self-assembly in water. Well-dispersed spheres investigated from TEM image took a shape from the polymer self-assembled into nanoparticles with the glyco block as the shell and the rod block as the core. These nanoparticles had the similar hydrodynamic radius between 19 and 38 nm with PDI around 0.21. These self-assembled nano-objects containing glycopolymers with well-defined isomerism on the surface showed some crucial properties such as high-water solubility, high stability during their binding to the specific lectins and the sustainability of multivalent binding tendency. The binding ability of these nanoparticles was tested with PNA and *Erythrina crista-galli* agglutinin (ECA). According to the results, even though NP-1-Man and NP-6-Gal did not show any binding ability with both PNA and ECA, NP-1-Gal showed strong binding with both lectins. Moreover, the asialoglycoprotein receptor (ASGPR) was used as a model of human lectin and its binding affinity with nanoparticles was examined by a quartz crystal microbalance (QCM). Expectedly, all nanoparticles showed similar binding ability with ASGPR due to previous investigations. This work is a very important proof to reveal how the effects of sugar regioisomersim in glycopolymers on their biological functions.

Muñoz-Bonilla et al. (2013) developed a very efficient approach to prepare a variety of amphiphilic block glycopolymers based on 2-{[(d-glucosamin-2-*N*-yl)carbonyl]oxy}ethylacrylate (HEAGI) and *n*-butyl acrylate (BA) or methyl methacrylate (MMA) by ATRP. They have reported the use of acrylic-based monomers and methacrylic glycomonomers on well-defined glycopolymers and presented an easy methodology to prepare amphiphilic glycopolymer. Firstly, unprotected glycomonomer (HEAGI) was synthesized according to procedure described previously. The monofunctional and difunctional macroinitiators of poly(BA) and poly(MMA) were prepared to use for the homopolymerization reaction of the glycomonomer carried out in DMF at 90°C by using *N,N,N*′*,N*′*,N*′-pentamethyldiethylenetriamine (PMDETA) and CuCl as a catalyst system. In this way, a small library of the well-defined amphiphilic block glycopolymers having di- and triblock glycopolymers with different hydrophobic blocks and varying the hydrophilic block lengths was demonstrated. These amphiphilic block glycopolymers showed the self-assembly ability to form micelles in aqueous solution. The dynamic light scattering (DLS) was used to determine the size of micelles at a copolymer concentration of 1 mg/ml at 25°C in distilled water or adding NaCl solutions. The DLS measurements revealed that the average diameters values are between 150 and 160 nm due to strong hydrogen bond interactions between hydroxyl groups and these polymers.

Moreover, their binding affinity with ConA was analyzed by using the turbidimetry. Interestingly, the results confirmed that the architecture of the block glycopolymers, diblock, or triblock glycopolymer did not show a significant influence on the lectin recognition process. It means that the glucose density over the polymer chain affects the molecular recognition independently in this study.

### Promoted self-assembly of glycopolymers

**Figure 11:**
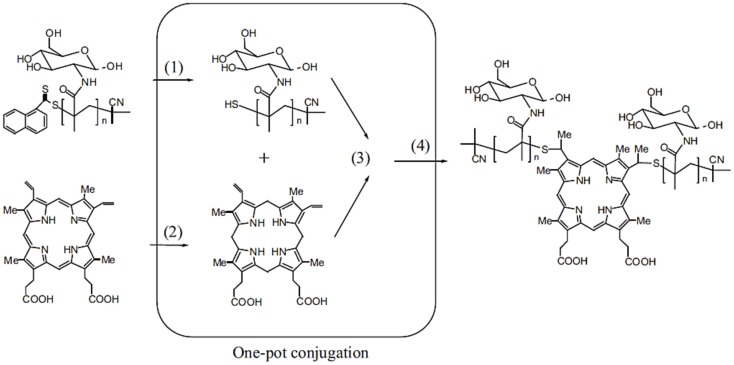
**Schematic presentation of one-pot synthesis of glycopolymer–porphyrin conjugate: (1) reduction of RAFT end group to thiols, (2) reduction of PpIX to propoporphyrinogen, (3) thiol–ene reaction of protoporphyrinogen with thiol-terminal glycopolymer, and (4) the oxidation of protoporphyrinogen to afford the porphyrin–glycopolymer conjugate**. Reproduced with permission from John Wiley and Sons, Copyright 2013 (Lu et al., 2014a).

An elegant one-pot system was developed by Lu et al. (2014a) for the synthesis of novel porphyrin–glycopolymer conjugates. RAFT polymerization and one-pot conjugation reaction were used together to combine multi-reactions. Firstly, methacrylamido glucopyranose (MAG) was polymerized via RAFT using 2-cyanoprop-2-yl-a-dithionaphthalate (CPDN) as the RAFT agent and AIBN as the initiator. Then, the obtained glycopolymers were conjugated with protoporphyrin (PpIX) via one-pot reaction combining the multi-step reactions including reduction of RAFT end group to thiols, converting protoporphyrin to protoporphyrinogen, thiol-ene reaction of protoporphyrinogen with thiol-terminal glycopolymer, and the oxidation of protoporphyrinogen to yield the porphyrin–glycopolymer conjugate, respectively. Sodium mercury amalgam was used as a catalyst during the one-pot reaction (Figure 11). All these following reactions were confirmed by NMR, UV–Vis, and fluorescence spectroscopy.

The obtained porphyrin-PMAG conjugates showed the amphiphilic behavior in the water due to hydrophobic porphyrin in the middle and hydrophilic glycopolymer at both ends. Scanning electron microscopy (SEM) and DLS were used to characterize the self-assembled micelles. Their binding abilities with ConA as well as anti-cancer effect for cancer cells (K562) were studied. Glycomicelles showed high and specific binding ability with ConA. In *in vitro* studies, the cytotoxic test of glycoparticles against K562 cells in low doses revealed that these self-assembled micelles killed the cancer cells under light irradiation and light treatment length dependent manners. Therefore, this report is quite promising for the development of applications for cancer imaging and therapy.

### Crosslinked glycopolymer capsules

**Figure 12:**
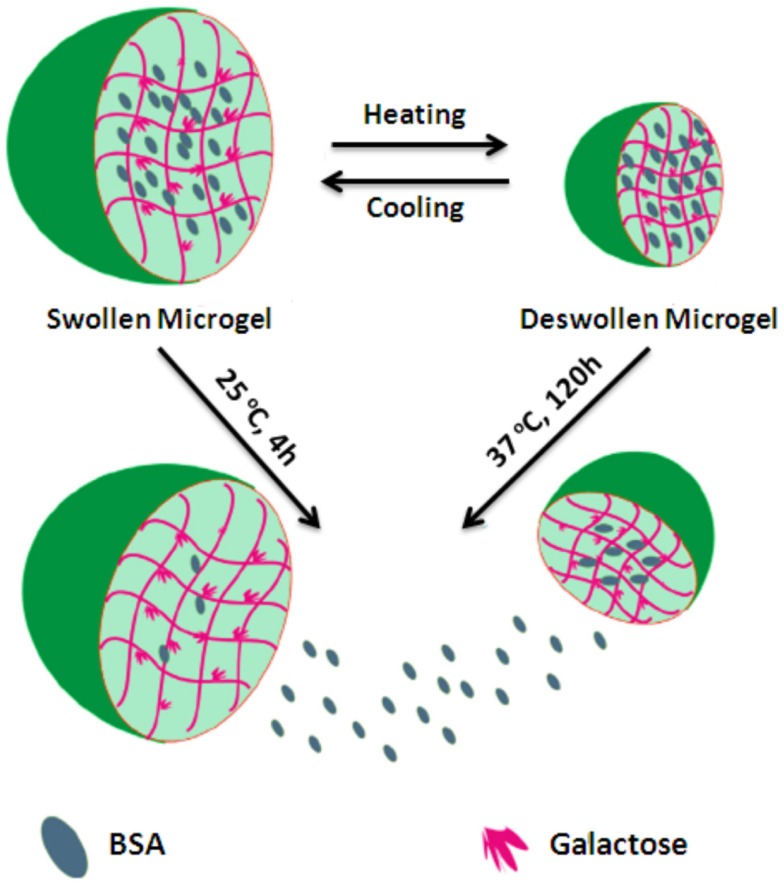
**Novel thermoresponsive microgels with tunable response profiles have been prepared and shown to have utility in the storage and release of BSA**. Reproduced with permission from Elsevier, Copyright 2014 (Lou et al., 2014).

Lou et al. (2014) achieved to produce novel galactose functionalized thermoresponsive injectable microgels. Poly(*N*-isopropylacrylamideco-6-*O*-vinyladipoyl-d-galactose) P(NIPA Am-*co*-VAGA) was synthesized via a combination of enzymatic transesterification and emulsion copolymerization. Firstly, 6-*O*-vinyladipoyl-d-galactose (VAGA) was prepared by controllable regioselective enzymatic transesterification with alkaline protease as a catalyst in anhydrous pyridine at 50°C for 4 days. The purification was performed by gel column chromatography. Subsequently, the free radical precipitation emulsion polymerization of NIPAAm with VAGA was performed in the presence of the crosslinker *N,N*-methylenebis(acrylamide) (BIS) and sodium dodecyl sulfate (SDS) in deionized water at 70°C using ammonium persulfate (APS) as an initiator to yield a series of galactose functionalized thermoresponsive crosslinked microgels. Field emission SEM and DLS were used to show the porous structure of microgels and a dramatic reduction in particle size when they were heated above their critical solution temperatures. The microgels became more viscous liquids that flowed easily and were injectable when they were heated up to body temperature. In *in vitro* study, the microgels were loaded with bovine serum albumin (BSA) and its release studied at 25 and 37°C (Figure 12). These results showed that the faster release rate of BSA was obtained below the LCST of the polymers.

This elegant report achieved the designing novel microgel drug delivery system that was the combination of themoresponsive and hepatocellular carcinoma targeting attributes into a single polymer. These novel thermoresponsive injectable microgels seem to have a potential for a wide range of biomaterials applications.

### Glycopolymer-grafted nanoparticles surface

**Figure 13:**
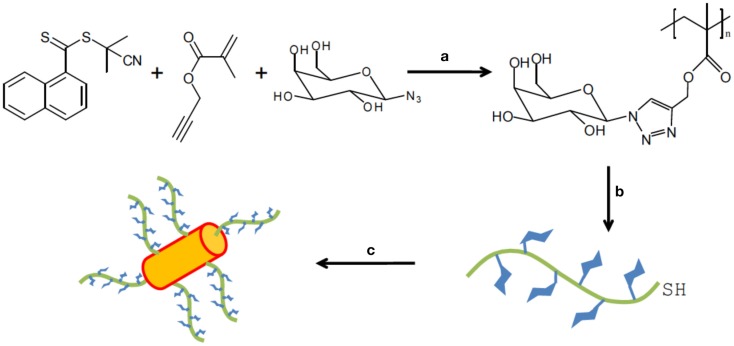
**Synthesis of polymer-coated gold nanoparticle**. Conditions: **(a)** synthesis of the glycopolymer with chain transfer agent at the end: copper powder, methyl 2-bromopropionate, DMSO, 25°C; **(b)** reduction of the end group of glycopolymer to a thiol: 2 equiv of R-NH_2_/NEt_3_, DMF, 50°C, 24 h; **(c)** reacting thiol-terminated glycopolymers with gold nanorods: gold nanorods, H_2_O, 12 h. Reproduced with permission from Royal Society of Chemistry, Copyright 2013 (Lu et al., 2014b).

The achievement for the preparation of the modified gold nanorods (GNRs) with glycopolymeric coatings was employed by Lu et al. (2014b) The Cu(0)-catalyzed one-pot reaction combining SET-RAFT for the synthesis of glycopolymers was investigated for the first time in this study. Side-chain functionalized glycopolymers were prepared via one-pot and one-step technique. The polymerization and click reaction were carried out *in situ* using 2-cyanoprop-2-yl-a-dithionaphthalate (CPDN) as the RAFT agent and EBBr as the initiator in DMSO at 25°C. Subsequently, PMDETA was added and the reaction mixture was kept for 4 h. The polymerization kinetics revealed that the relationship between the molecular weight and the monomer conversion was linear with narrow polydispersity (Mw/Mn = 1.1–1.3). Therefore, this approach provided a design of polymers with special side-chain functionality. Moreover, the rate of click reaction was significantly higher than the polymerization rate. In order to make the glycopolymers being grafted to gold nanrods easily, the end-group reduction of the glycopolymers was undertaken in the presence of hexylamine/triethylamine as reductant at 50°C for 24 h. The thiol-terminal groups were confirmed by UV–Vis spectroscopy after the end-group modification. Then, these thiol-terminated glycopolymers covered the surface of gold nanorods to form a self-assembled monolayer on the GNPs surface due to the interaction of Au–S bond (Figure 13). The obtained glyco-nanorods were examined via TEM and DLS. According to the selective binding ability of PNA with galactosyl residues, PNA was used a model lectin to study the recognition ability of the nanoparticles with lectins. The results demonstrated that the glycopolymer substituted GNRs have a great binding affinity with PNA due to the sufficient numbers of galactose groups on the surface of the nanoparticles. This work opened new avenue for polymer chemists to prepare well-defined glycoparticles by using this one-pot/one-step technique.

Lu et al. (2014c) have recently been able to prepare glycopolymer-functionalized Ag nanoclusters (Gly–Ag NCs) that were fluorescent and presented high-binding ability and cytotoxity. RAFT polymerization was employed for the synthesis of sugar- and acid-containing polymers in the presence CPDN as the RAFT agent and AIBN as the initiator in DMF at 70°C. Then, these glycopolymers were mixed with AgNO_3_ and then placed into the CEM instruments. Silver nanoclusters that are decorated with glycopolymers (PMAG-Ag NCs) were fabricated in the absence of reducing agents under microwave irradiation. Silver nanoclusters were chosen to decorate the glycopolymers due to their fluorescent and cytotoxic properties providing advantages in both cancer imaging and therapy. These nanoclusters were characterized using SEM, TEM, AAS, and fluorescence spectroscopy. These fluorescent nanoparticles could significantly bind to and show high cytotoxicity against GLUT over-expressing cancer cells K562. Moreover, they inhibited their viability possibly through the enhancement of reactive oxygen species (ROS) production. Therefore, the obtained nanoclusters are promising to treat cancers, such as multidrug-resistant tumors and eye-related neo-vascular diseases.

### Glycopolymers as targeted gene delivery vehicles

Kurtulus et al. (2014) developed a gene delivery system via the synthesis of spermine-like glycopolymer using RAFT. They reported for the first time to synthesize a new methacrylate monomer, namely, 2-((tert-butoxycarbonyl) (2-((tert-butoxycarbonyl)amino)-ethyl)amino)ethylmethacrylate (BocAEAEMA). After the RAFT polymerization of BocAEAEMA, P(BocAEAEMA) was used as a macro-RAFT agent to make chain extension with mannose acrylate (ManAc) synthesized in according to the procedure reported by Roy and Mukhopadhyay (2007) This chain extension reaction was undertaken in DMF at 70°C for 12 h using AIBN. After the deprotection of amine groups by removing BOC groups, P(AEAEMA)-b-P(ManAc) was obtained to analyze the binding ability of this well-defined glycopolymer with DNA. Even though P(AEAEMA) showed high-proton sponge capacity in comparison to polyethyleneimine (PEI) to form polyplexes with DNA because of electrostatic force between polymer and DNA, the glycopolymer block decreased this interaction between P(AEAEMA) and DNA regardless of their specific binding affinity with proteins. Besides, the size of P(AEAEMA)-b-P(ManAc) and DNA formed polyplex particles are found to be smaller than the size of P(AEAEMA) and DNA formed particles in according to DLS measurements due to the hydrophilic property of the glycopolymer. These synthesized spermine-like glycopolymers can be used as endosomal escaping agents for the applications of several treatments, such as gene therapies.

**Figure 14:**
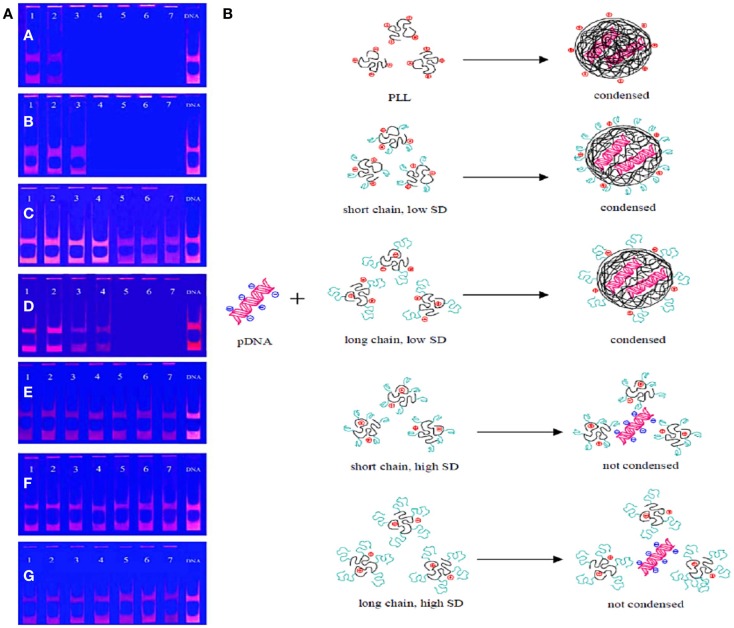
**(A)** pDNA condensation by the p-PLL and the DPMAEL-*g*-PLL. (a) The p-PLL is mixed with the pDNA and incubated for 30 min, the N/P (charge ratio of amine to phosphate) ratios corresponding to lanes 1–7 are 0.3125, 0.625, 1.25, 2.5, 5, 10, 20; (b,c) the DPMAEL-*g*-PLL-1 or DPMAEL-*g*-PLL-2 is mixed with the pDNA and incubated for 30 min, the N/P ratios corresponding to lanes 1–7 are 0.4, 0.8, 4, 8, 16, 24, 32; (d,e) the DPMAEL-*g*-PLL-3 or DPMAEL-*g*-PLL-4 is mixed with the pDNA and incubated for 30 min, the N/P ratios corresponding to lanes 1–7 are 0.2, 0.4, 1.6, 4, 8, 12, 16; (f) the DPMAEL-g-PLL-2 is mixed with the pDNA and incubated for 24 h, the N/P ratios corresponding to lanes 1–6 are 16, 24, 32, 40, 48, 64, 80; (g) the DPMAEL-*g*-PLL-4 is mixed with the pDNA and incubated for 24 h, the N/P ratios corresponding to lanes 1–6 are 16, 24, 32, 40, 48, 60, 80. **(B)** Illustration of the influences of the glycopolymer length and SD (substitution degree) on the pDNA condensation by PLL. Reproduced with permission from Elsevier, Copyright 2012 (Zhou et al., 2012).

Zhou et al. (2012) reported a strategy for gene delivery system based on the synthesis of well-defined glycopolymers grafted onto Poly(l-lysine) (PLL). Despite of the significant condensation capacity with plasmid DNA due to positively charged hydrophilic amino group in water, PLL is not generally chosen as gene delivery vectors because of high cytotoxicity and low-transfection efficiency. In this study, to reduce the cytotoxicity and increase the transfection efficiency of PLL significantly, PLL was modified by well-defined saccharide-containing polymers. Firstly, glycomonomer, 2-*O*-meth-acryloyloxyethoxyl-(2,3,4,6-tetra-*O*-acetyl-β-d-galactopyranosyl)-(1–4)-2,3,6-tri-*O*-acetyl-β-d-gluco pyranoside, (MAEL) was polymerized via RAFT in the presence of CPADB and AIBN in chloroform at 70°C for 24 h. After the deprotection of the acetyl groups, the obtained glycopolymers were grafted onto PLL through the amidation reaction between the carboxyl group of the dithioester residue on the glycopolymer and the amino group on PLL mediated NHS and 1-Ethyl-3-(3-dimethylaminopropyl) carbodiimide (EDC) to yield four different DPMAEL-*g*-PLL due to their different polyelectrolyte properties because of the N/P (charge ratio of amine to phosphate) ratios. The zeta potentials of these DPMAEL-*g*-PLL complexes were measured by DLS and an increase of the N/P ratio turned the zeta potentials to positive charge. The glycopolymer modified PLL showed lower cytotoxicity than PLL in according to the MTT assays with the mouse embryonic fibroblast cell line (NIH3T3) and human hepatoma cell line (HepG2). Moreover, these obtained complexes enhanced the transfection efficiencies of PLL, as well. As depicted in Figure 14, pDNA condensation by the DPMAEL-g-PLL complexes was investigated by agarose gel electrophoresis. The results demonstrated that low SD (substitution degree) on PLL increased the pDNA condensation capacity. This work allowed PLL to be more useful and applicable for gene delivery with enhanced biological properties.

### Electrospinning of glycopolymer fibers

**Figure 15:**
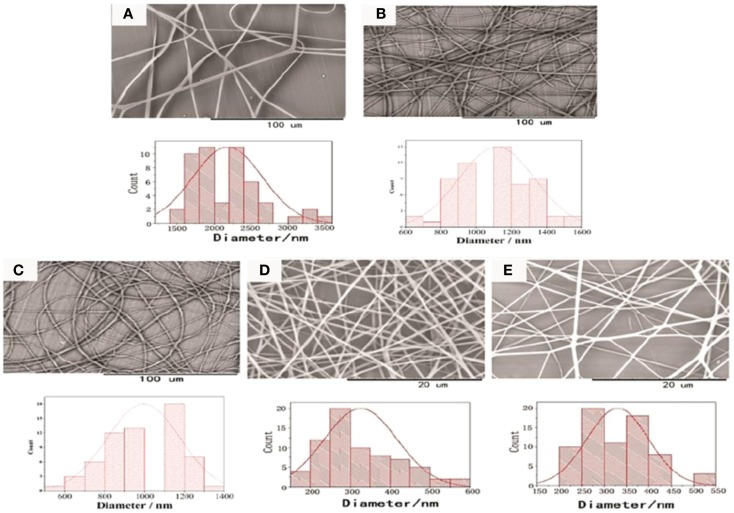
**Scanning electron microscopy images of the electrospun fibers prepared in this work (A) PNIPAM/PLCL; (B) PND-1-A; (C) PND-1-B; (D) PND-2-A; (E) PND-2-B**. Reproduced with permission from Royal Society of Chemistry, Copyright 2014 (Wang et al., 2014b).

Wang et al. (2014b) employed to synthesize the thermoresponsive glycopolymers poly(*N*-isopropyl-acrylamide-*co*-6-*O*-vinyladipoyl-d-glucose) (poly-NIPAM-*co*-OVDG; PND) and poly(*N*-isopropylacrylamide-*co*-6-*O*-vinylazelaicoyl-d-glucose) (poly-NIPAM-*co*-OVZG; PNZ) via free radical polymerization process. The polymerization reaction was undertaken in anhydrous ethanol at 60°C for 8 h with the presence of AIBN as an initiator. In total, a set of six copolymers was prepared with different ratios of NIPAM. Then, electrospinning process was used to prepare nanofibers comprising blends of poly-NIPAM-*co*-OVDG/poly-NIPAM-*co*-OVZG with poly-l-lactide-*co*-ε-caprolactone (PLCL). Before electrospinning process, the spinning solutions were prepared by dissolving of PND/PNZ and PLCL in a mixture of dichloromethane and acetone (2:1 v/v) at ambient temperature and stirring until forming a homogeneous solution. An electrical potential of 12 kV was applied while the spinning solution was flowing through a stainless steel capillary needle to a collector plate coated in Al foil. As depicted in Figure 15, the obtained nanofibers were characterized by using SEM. Additionally, MTT assay confirmed that these nanofibers have generally good biocompatibility with HeLa cells and minimum cytotoxicity, as well. Even though these nanofibers did not have sufficient ability to inhibit non-specific adsorption of bovine serum albumin onto their surfaces, they showed significant interaction with ConA. The results revealed that when these glycopolymer nanofibers are loaded with ConA, the adsorbed ConA can easily be desorbed with a glucose solution and used to induce death in the cell population. This work can be utilized easily into other glycosylated polymers given their temperature sensitive properties and have the sensitive and specific recognition with lectins.

## Conclusion and Outlook

In this review, the synthesis of the various well-defined glycopolymer/particles architectures and their interaction with corresponding lectins were discussed to provide readers a general overview on the creation of different bioactive glycopolymer structures for various health related applications such as drug delivery, biomaterials, bio- and nanotechnologies, and gene therapy. Very recent and elegant synthetic routes have allowed polymer chemists to prepare a wide range of glycopolymers and glyconanomaterials that exhibit excellent and significant recognition properties toward lectins. These studies bring us a step closer to being able to establish the glycopolymer code and to prepare complex glycopolymer architectures as a glycan mimic. The ability of the investigated glycoparticles in the nanometer scale to mimic the behavior of naturally existing glycocalyx has showed promising results in the design of interfaces with different chemical functional groups. Moreover, self-assembled glyconanostructures with a broad variety of morphologies were highlighted for their potential as an alternative route for the systemic delivery of drugs in the treatment of diseases such as hepatitis C, cancer, and others. In summary, all these novel methodologies on the synthesis and investigation of well-defined glycopolymers have a great potential for future studies to further enhance their specific recognition properties and to develop therapeutic agents and biological probes. It is envisioned that in the next couple of decades interdisciplinary approaches on the designed glycopolymer or glyconanomaterial synthesis and on the medical or pharmaceutical applications for therapeutic purposes will be revolutionizing the medical treatments of patients.

## Conflict of Interest Statement

The authors declare that the research was conducted in the absence of any commercial or financial relationships that could be construed as a potential conflict of interest.
